# Dynamic behavior of suspending nanodroplets with coming nanodroplets on solid surfaces: A molecular dynamics study

**DOI:** 10.1371/journal.pone.0334956

**Published:** 2025-11-07

**Authors:** Liwei Sun, Xiaochun Pan, Jiachao Gu, Baocheng Zhan

**Affiliations:** School of Mechanical Engineering, Changchun Technical University of Automobile, Changchun, China; IGDTUW: Indira Gandhi Delhi Technical University for Women, INDIA

## Abstract

The impingement of binary droplets upon solid surfaces has received growing attention in recent years because of its wide applications in industry. Although the dynamic evolution of impacting single droplets is relatively well satisfied, the understanding of impacting binary droplets remains inadequate, especially at the nanoscale. This paper uses molecular dynamics (MD) simulations to investigate the impact behavior of suspending nanodroplets with coming nanoscale ones to address this gap. The wettability of solid surfaces and the impact *We* are selected in a wide range to obtain an overall insight into the impingement of targeted systems. Firstly, the representative dynamics are recognized from snapshots to observe the corresponding instantaneous evolution at the molecular level. Secondly, the maximum spreading of impacting binary droplets during coalescence (primary spreading) and extension upon solid surfaces (secondary spreading) is investigated. Finally, the contact time of targeted systems is discussed by extracting data from numerical simulations over a wide range of *We* and intrinsic contact angles. The present work can provide a guideline for the application of impacting multiple nanodroplets, which requires a controllable spreading diameter or quick detachment of impacting nanodroplets.

## 1. Introduction

Impingement of droplets on solid surfaces is one of the most fascinating phenomena in interfacial science due to its wide application in industrial processes, for example, spray cooling [1,2], wing anti-ice [3,4], droplet transport [5,6], surface self-cleaning [7,8], and inkjet printing [9–11]. Therefore, this topic has been received growing attention in recent years. Since the first work focused on impacting phenomena [12], many theoretical analyses and experimental tests have been performed to investigate impacting droplets together with dynamic behaviors [13,14]. Generally, the spreading, retracting, and deposition during droplets’ impinegment take place successively after droplets interact with solid surfaces [15–17].

The dynamic evolution of impacting droplets is a consequence of combined action between several competing forces, typically including inertial, capillary, and viscous forces [18,19]. Additionally, the physical property of impacting droplets, such as viscosity, density, and surface tension, can also affect impacting processes [20,21]. Therefore, the impingement of droplets involves complex physical phenomena, and thus, several dimensionless parameters are proposed to simplify the investigated systems [22,23]. There are two representative parameters, Weber number (*We* = *ρD*_0_*V*_0_^2^/*γ*) and Reynolds number (*Re* = *ρD*_0_*V*_0_/*μ*) [24,25], which represent the ratio of inertial force to capillary force and the ratio of inertial force to viscous force. Here, *ρ*, *γ*, and *μ* are the density, surface tension, and viscosity of the droplet, *V*_0_ is the impacting velocity, and *D*_0_ is the diameter of the impacting droplet. There are two extreme conditions during droplets’ impingement, i.e., viscous and capillary regimes, which can be distinguished by different dominant forces [26–28]. The viscous regime corresponds to such a situation where *Re* is much larger than *We*, and thus, the capillary force can be neglected safely. In contrast, for the capillary regime, the effect of the viscous force is out of consideration (*We*>>*Re*). For the topic of water droplet impact, the maximum spreading diameter is one of the most important characteristic parameters because it is essential for high-precision applications [29]. The maximum spreading diameter is often normalized by the so-called maximum spreading factor, expressed as *β*_max_ = *D*_max_*/D*_0_. Herein, the *D*_max_ is the diameter of impacting droplets at the maximum spreading state. Importantly, the parameter of *β*_max_ can not only guide design of printing devices but also reveal relationship between kinetic energy and surface energy [30]. The kinetic energy converts to the surface energy when impacting droplets start to spread, and the subsequent progress attains the maximum spreading state. The undergoing retraction stage is just against the spreading stage, where the surface energy returns back to the kinetic energy again. The previous work demonstrated that, for the viscous regime, the energy loss during spreading processes is mainly caused by viscous dissipation [28]. The author also noted that the maximum spreading factor can be predicted using a law of *β*_max_ ~ *Re*^1/5^ [28]. On the contrary, the maximum spreading factor’s law at the capillary regime follows *β*_max_ ~ *We*^1/4^, which agrees well with experimental observations for low-viscosity fluid [26]. There is a crossover regime between viscous and capillary regimes where the *β*_max_ is controlled by a coupling effect between viscous force and capillary force. It is very difficult to predict the relevant *β*_max_ for cross-over regimes because the relationship between *β*_max_ and *We* and *Re* is very complex and is changeable.

Recently, the impingement of nanoscale droplets has stimulated a great advancement in nanotechnologies, such as nanoscale spray cooling and nanoscale coating [31–33]. Therefore, the topic on impacting nanoscale droplets has received growing attention. Conventional experimental techniques face significant challenges in observing the dynamic evolution of the nanoscale droplet because of limitation of resolution of optical microscopes. In contrast, molecular dynamics (MD) simulations have proven to be an effective tool for studying the impingement behavior of microscale droplets ranging from tens of meters to tens of nanometers [34,35]. Although the dynamic evolution of impapcting a single nanodroplet is relatively well understood, studies on the impingement of multiple droplets remain scarce. Actually, the impingement of multi-droplet systems should be the most common scene in practical applications, such as electronic packaging, rapid prototyping, and so forth. Moreover, the impingement of multiple droplets is undoubtedly more complicated, which could induce some novel dynamics. Collisional macrodroplets can be separated from each other again to form temporary coalescence, which is a consequence of liquid bridge being destroyed by surplus surface energy and only occurs at a very large *We* range [36,37]. Otherwise, the collisional process can cause continuous drainage of air film, and eventually, this film can be ruptured to form a secure liquid bridge, i.e., permanent coalescence [38]. However, the permanent coalescence is an exclusive result as size of collisional droplets is reduced to the nanometer scale. Ref. [39] explicitly demonstrated that temporary coalescence can be attributed to an enhanced role of surface forces, stemming from the significant increase in the surface-to-volume ratio.

To date, several important scale effects have been identified for impacting nanodroplets. One example is that, for impacting droplets at the nanoscale, the ratio of surface area to volume can be significantly increased, i.e., the increasing surface force. Another significant example is that the Ohnesorge number, *Oh* = *μ*/(*ρD*_0_γ)^1/2^, increases from *O* (10^−3^) to *O* (1), indicating that the viscous force must be considered even for low-viscosity fluid. Xie et al. [39] demonstrated that the contact time, *t*_c_, for low-viscosity nanodroplets (such as water and argon) follows a new scaling law of (*D*_0_/*V*_0_)*We*^1/2^*Oh*^1/3^, which also confirms the enhanced viscous effect in nanoscale impingement. Here, the contact time is defined as the period from a droplet just touching to bouncing off from a surface.

The present work aims to examine the dynamic characteristics of impacting a suspended droplet with an incoming droplet on surfaces ranging from hydrophilic to superhydrophobic via MD simulations. The *We* is varied over a range of 0.3-300, consistent with numerous previous studies [5,31,39]. Representative behaviors of impacting binary droplets at the nanoscale have been observed. The impingement typically involves coalescence into a larger droplet (primary spreading), followed by conventional spreading on the surface (secondary spreading), retraction, and potentially rebound. After that, this paper mainly explores the maximum spreading factor, *β*_max_, of impacting binary droplets, including the primary spreading and secondary spreading, and attempt to establish the relationship between the maximum spreading factor and the impact *We* for the primary spreading and the secondary spreading. Finally, the paper discusses the variation of the contact time under different given conditions. Interestingly, impacting binary droplets can achieve rapid detachment, surpassing the well-known theoretical minimum contact time limit. This finding is expected to benefit applications requiring enhanced self-cleaning and superhydrophobic performance.

## 2. Simulation method

**Fig 1:**
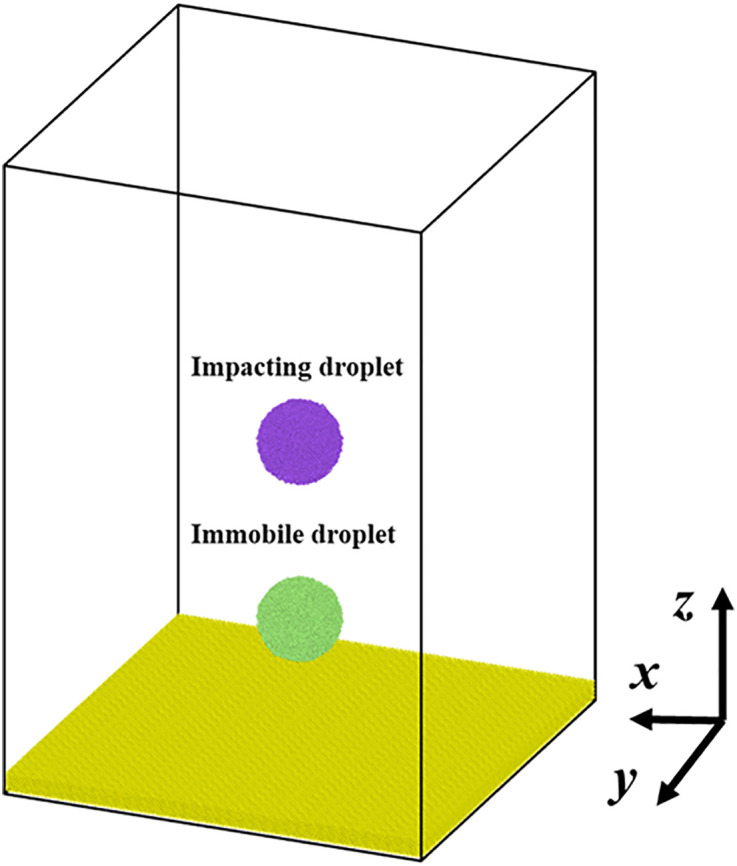
Simulated configuration which contains two identical nanodroplets with radius of 4 nm and Pt solid surface are placed. One of which is in a suspending state and another one is impacting droplet with different velocity.

Here, the paper chooses MD simulations (LAMMPS) to study the dynamic behavior of the nanoscale impingement of a head-on droplet with a coming one. The initial configuration of the MD system is illustrated in **Fig 1**, which contains an impacting droplet, an immobile one suspended in the simulated box, and a solid surface comprised of Pt atoms. Two droplets are identical, and each one contains 8900 water molecules, and the corresponding radius of each droplet is 4 nm. The thickness of the Pt surface is 1.97 nm, containing 185000 Pt atoms. The center-of-mass coordinates of the two nanodroplets are (0, 0, 12) and (0, 0, 29), respectively, and hence the separating distance between the collisional droplets is 17 nm. Artificial virtual springs are applied to constrain the metal atoms to their equilibrium positions, preventing deformation, as described in previous works [40,41]. The dimensions of the simulated box in the *x*-, *y*-, and *z*- directions are 40 nm, 40 nm, and 60 nm, respectively, which is sufficiently large enough to observe the dynamic evolution of impacting nanodroplets.

The mW water model is selected to describe the intermolecular interactions between water molecules. Compared with the other water models, such as TIP4P and SPC/E models, the mW water model can greatly reduce the simulated cost because it is a monatomic water model [42–44]. Even so, the physical properties of the mW model still can be accurately reproduced, for instance, the density of the model is *ρ* = 0.997 g cm^−3^ and the surface tension is *γ*_lv_ = 66 mN m^−1^, which is very close to real water [45]. Owing to the omission of the hydrogen atom reorientation, the viscosity of the model is *μ* = 283.7 μPas^-1^, three times lower than the experimental value [46]. The intermolecular force for both water-Pt and Pt-Pt is described by the Lennard-Jones 12−6 potential, expressed as


$$U_{LJ}\left(r\right)=4\varepsilon \left[{\left(\frac{\sigma }{r}\right)}^{12}-{\left(\frac{\sigma }{r}\right)}^{6}\right],r<r_{cut}$$ (1)

where *r* is the distance separating two adjacent atoms, *ε* is the depth of the potential wall, *σ* is the zero-crossing distance, and *r*_cut_ is the cutoff distance with a value of 1nm, which was widely used before [47]. The interaction between water molecules is described by parameters of *ε*_w_ = 0.26838 eV and *σ*_w_ = 0.23925 nm, whose values change to *ε*_P_ = 0.69375 eV and *σ*_P_ = 0.247 nm to describe Pt-Pt interaction. Previous studies demonstrated that these parameters are effective in describing the dynamic behavior of nanoscale droplets, including impingement of nanodroplets, wetting transitions of nanoscale fluids, and spontaneous jumping of coalescence nanodroplets.

Subsequently, the system is equilibrated for 1 ns with a time step of 1 fs to obtain the equilibrium state. To achieve this purpose, the system is run in the NVT ensemble at 300 K over a pre-equilibrium process using the Nose–Hoover thermostat [48,49]. Subsequently, two droplets are run in the NVE ensemble for another 1 ns in productive processes. Impacting droplets are endowed with a series of vertical velocities to impact suspending ones in an attempt to observe the dynamic evolution from coalescence, spreading, and retraction to bounce, and break up. The wettability of solid surfaces is generally controlled by a parameter of *ε*_w-Pt,_ which can express the interaction between water and surfaces. Here in this paper, a wide range of *ε*_w-Pt_ from 0.0136 eV to 0.0018 eV is chosen to construct different intrinsic wettability from hydrophobic to superhydrophobic.

## 3. Results and discussion

**A. Temporal evolution of targeted systems**

**Fig 2:**
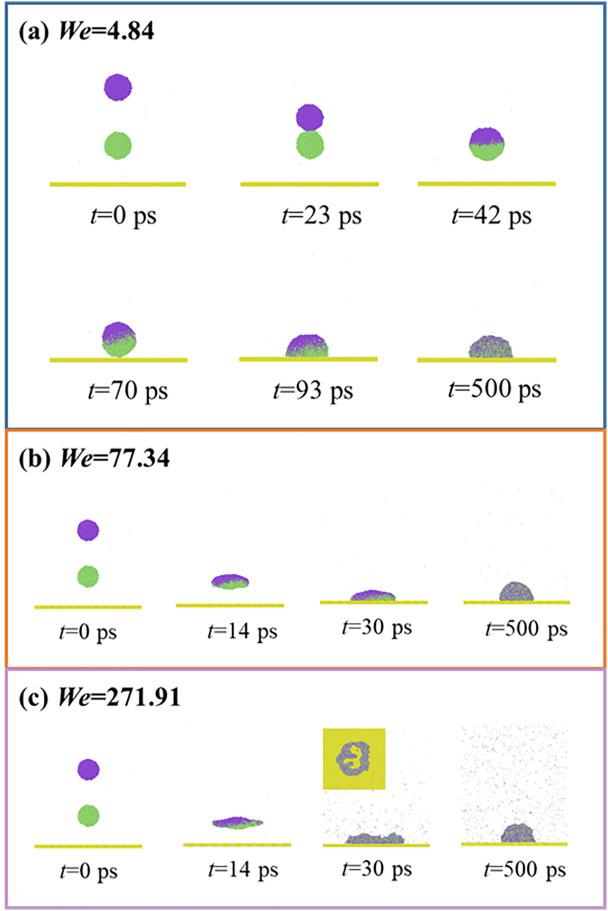
Dynamic evolution of impacting binary nanodroplets on a hydrophohilic surface with *θ*_Y_ = 85° at (a) *We* = 4.84, (b) *We* = 77.34, and (c) *We* = 271.91.

To investigate the representative dynamic process, The dynamic behavior of impacting binary droplets is first presented for different *We* and the intrinsic wettability is initiated with *θ*_Y_ = 85°, as illustrated in **Fig 2**. At *We* = 4.84, the low-velocity impingement shows that the impacting droplet moves slowly towards the immobile one, and they come into contact with each other at *t* = 23 ps. Subsequently, the two separated droplets coalesce into a merged droplet via a liquid bridge driven by the pressure difference. The growth of the liquid bridge results from the coupled effects of Laplace pressure and inertial forces. The process of the liquid-bridge expansion is regarded as primary spreading in the topic of impacting binary droplets, as described in Ref. [50]. During this part, the kinetic energy of the droplet is converted into surface energy needing for the growth of the bridge. The merged droplet adopts a spherical shape of a sphere at low *We* impingement and continuously descends towards the surface after primary spreading is complete. The merged droplet contacts with the solid surface after 70 ps and begins to experience secondary spreading, analogous to the common spreading of an impacting single droplet (*t* > 70 ps, **Fig 2a**). The merged droplet attains its maximum spreading state, followed by retracting to a stable deposited droplet till the end of the simulation (see 500 ps). Due to the involvement of the solid surface in the impacting process, the motion of the merging droplet is subjected to a combination of inertial, capillary, and viscous forces, the droplet viscous effect is enhanced, and the horizontal velocity gradient induced by the solid surface triggers additional dissipation. When *We* increase to 77.44, the impacting droplet quickly moves and induces a dramatic primary spreading to form a pancake-like spreading state, as shown at *t* = 14 ps in **Fig 2b**. The secondary spreading allows *t*he pancake-like liquid to further extend and become a extremely thin spreading film (see 30 ps). The released surface energy stored in droplet extension triggers the droplet retraction to be a wettable droplet, like low-velocity impingement. On further increasing *We*, there is a very violent impacting process in both primary and secondary spreading, as shown at *We* = 271.91 in **Fig 2c**. The merged droplet can not endure the continuous deformation over the secondary spreading and forms a huge hole within the spreading film (from the top view in the insert picture), i.e., the hole spreading of impacting binary droplets. Under the action of a surface force from retracting the droplet, this hole closes up again. The final wettable droplets for all three cases should be equal and show an independent feature of the impacting velocity. The wettable water droplet is in an equilibrium state between cohesion and liquid-solid interaction. Cohesion is an intrinsic feature of the wettable droplet, and liquid-solid interaction depends on the wettability of solid surfaces. Therefore, the final state of wettable droplets is only determined by the surface wettability, which is responsible for the reason why the same wetting states can be observed. Notably, within the investigated systems, the centroid of the merged droplet remains nearly stationary during primary spreading, no matter how *We* increase. This indicates that the energy is initially consumed by droplet deformation rather than used for inducing the movement of merged droplets. Therefore, for the primary spreading, the energy conversion for droplets’ extension is a preferential process compared with the movement of merged droplets.

**B. Spreading information over spreading**

**Fig 3:**
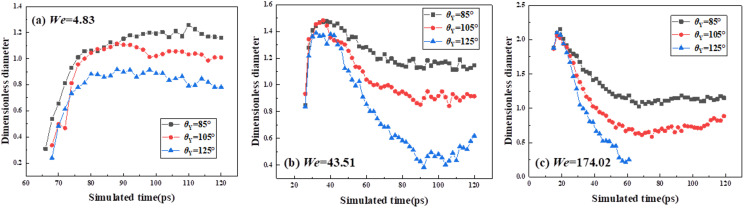
Variation of dimensionless spreading factor versus simulated time starting from the secondary spreading at various *θ*_Y_ and *We.*

**Fig 4:**
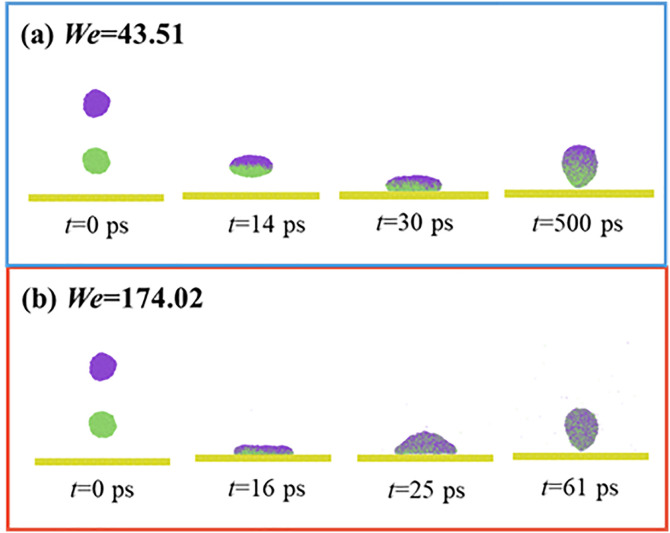
Snapshots of impacting binary droplets on a superhydrophobic surfaces with *θ*_Y_ = 125° at *We* = 43.51 and 174.02.

Previous studies commonly employ the variation of the dimensionless spreading factor, *β*, under different conditions to investigate impacting dynamics. In this work, varying *We* value should not show any effect on the primary spreading, so that the maximum spreading factor of the primary spreading, *β*_max, pre_, only depends on the impact *We*. Hence, the wettability only starts to affect spreading dynamics after merged droplets experience the secondary spreading. The paper extracts the variation of *β* as a function of *t* at various *θ*_Y_ and *We* to investigate their effect on impacting dynamics for targeted systems. The dimensionless spreading factor in this section starts from the secondary spreading, expressed as *β* = *D*_sec_/*D*_co_, where *D*_co_ is the diameter of the resultant droplet based on the controllable-volume method. The value of *D*_co_ is 10.08 nm, and *D*_sec_ is the spreading diameter at the initial state of secondary spreading. As shown in **Fig 3a**, the spreading rate for *θ*_Y_ = 85° and 105° is obviously higher than that for impingement at *θ*_Y_ = 125°, which results in a higher value of *β*_max, sec_. The process of droplets’ impingement is a consequence of the competitive relation among inertial, capillary, and viscous forces. It indicates that the impingement is in the capillary regime. Therefore, the process of droplet spreading is controlled by a couple of effects between inertial and capillary forces. The rest of the inertial force in moving merged droplets induces the secondary spreading, while the capillary force prevents merged droplets from further spreading on surfaces, especially for the solid surface with *θ*_Y_ = 125°. As a result, at the low range of *We*, the *θ*_Y_ can affect impacting processes in terms of spreading rate, and the decreasing *θ*_Y_ promotes spreading and results in a large *β*_max, sec_ value. Retraction is not observed at such low *We*, i.e., the maximum spreading state is very close to the equilibrium wetting state, and an increase in *θ*_Y_ promotes an increase in the wettable area. As shown in **Fig 3b** and **3c**, on further increasing *We* to 43.51 and 174.02, the *θ*_Y_ gradually loses its ability to control the spreading of targeted systems. The spreading rate is equal regardless of varying *θ*_Y_, reaching the same *β*_max, sec_ value, because the inertial force is too large to ignore the capillary effect. Although the capillary force can not affect the spreading stage, the capillary force is of great importance in altering the retracting dynamics. From **Fig 3b** and **3c**, the capillary force is found to promote the retraction of merged droplets, and thus, the retracting rate on the solid surface with *θ*_Y_ = 125° is the fastest between these three cases. At *We* = 43.51 and 174.02, a turning point is observed in the retraction process of the merged droplet at *θ*_Y_ = 125°. For example, the value of *β* could increase again after 90 ps in **Fig 3b**. The abnormal variation of *β* value indicates that the wettable state is no longer the equilibrium state, and the corresponding dynamics on surfaces with *θ*_Y_ = 125° are shown in **Fig 4**. The snapshots exhibit a continuous retracting process with reducing wettable areas, and eventually the merged droplets leave from the solid surface after they undergo the primary and secondary spreading, forming bouncing pattern (**Fig 4a**). Notably, the bouncing at high *We* = 174.02 exhibits non-classical behavior, which initially forms convex interface and subsequently recovers an elongated bouncing droplet at *t* = 61 ps, as shown in **Fig 4b**).

**Fig 5:**
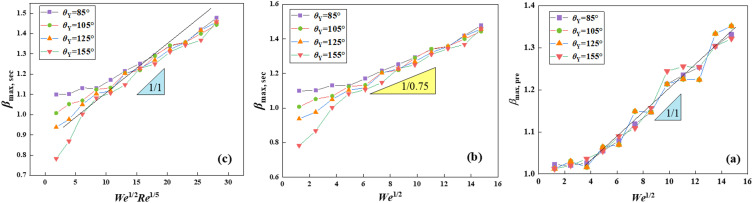
Maximum spreading factors of impacting binary droplets for both (a) *β*_max, pre_ and (b) *β*_max, sec_ on solid surfaces with various *θ*_Y_ at *We* = 43.51 and 174.02 as a function of *We*^1/2^.

After that, the paper focuses on the maximum spreading factor for both *β*_max, pre,_ and *β*_max, sec_ during different given conditions. As shown in **Fig 5a**, the *β*_max, pre_ is observed to be perfectly overlapped for each case and is independent of surface wettability, which confirms our speculation before. At low *We*, the deformation of the merged droplet is minimal. While some surface energy is released upon coalescence, it is insufficient to drive significant spreading. At a high range of impact *We*, only a part of the kinetic energy is used to convert to the surface energy, leaving some energy to induce the movement of merged droplets. For the nano-impingement, the impacting velocity can run through the whole impacting droplets, and the vortex motion for macrosystems vanishes, which leads to reduced energy consumption. By applying the principle of energy conservation, the initial kinetic energy to surface energy yields a relationship as *γ*_lv_ *β*_max, pre_^2^ ~ *E*_K_*V*^2^. Where *D*_max, pre_ is the diameter of the coalescing droplet at the previous maximum spreading state, and *V* is the initial velocity of the impact droplet. By introducing the formula for *We*, the *β*_max, pre_ is derived as follows a law of *β*_max, pre_ ~ *We*^1/2^. The variation of *β*_max, pre_ versus *We*^1/2^ can be fitted on a straight line with a prefactor of 1, except for the low *We* range, as shown in **Fig 5a**. The spreading conditions are found to vary after impacting merged droplets start to interact with solid substrates, as shown in **Fig 5b**. At a low range of *We*, the maximum spreading factor for *β*_max, sec_ is in inverse proportion to surface wettability due to the capillary force playing a non-negligible role, as described above. On further increasing *We*, the inertial force is dominant, leading to almost the same *β*_max, sec,_ regardless of varying *θ*_Y_. But the *β*_max, sec_ is not directly proportional to *We*^1/2^, having a prefactor of 0.75. This indicates that there is another force introduced during the secondary spreading on solid surfaces. To emphasise, the dynamic behavior on surfaces, the surface acts as an obstacle and water molecules within droplets may generate an obvious velocity gradient in the horizontal direction, leading to extra energy dissipation [46]. Meanwhile, the viscous force becomes a significant influencing factor at the nanoscal, leading to the dissipation of kinetic energy through the formation of viscous dissipation, which scales as *Re*^1/5^ as described in Ref. [13]. Consequently, *β*_max, sec_ should be governed by a relationship that combines *We*^1/2^ and *Re*^1/5^, resulting in the following relationship of *β*_max, sec_ ~ *We*^1/2^*Re*^1/5^. According to the analysis, this paper uses *We*^1/2^*Re*^1/5^ to replace the abscissa to depict a variation of *β*_max, sec_ again, as shown in **Fig 5c**. The prefactor modifies from 0.75 in **Fig 5b** to return to 1 after considering the effect of the viscous force over secondary spreading.

**C. Variation of contact time**

**Fig 6:**
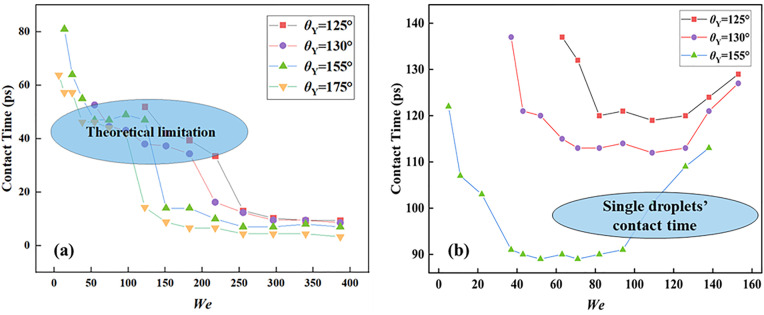
(a) Variation of contact times as a function of impact *We* on solid surfaces with various Young contact angles ranging from *θ*_Y_ = 125° to *θ*_Y_ = 175° and (b) Variation in contact time of single droplets of equal volume under the same conditions.

Next, the section focuses on the variation of contact time, *t*_c_, for impacting binary nanodroplets, which only becomes possible for impingement on superhydrophobic solid surfaces. For impacting binary nanodroplets, the contact time is defined as the time difference value between the two impacting dynamics. One is selected at the time point when the merged droplet first contacts a solid surface, and another one is when the merged droplet just leaves it. Achieving rapid detachment of impacting droplets from solid surfaces represents a significant current research challenge, because the shortened *t*_c_ is quite necessary in a variety of practical applications, such as anti-icing and self-cleaning [51]. For targeted systems, the bouncing behavior occurs when the Young contact angle increases over 125°. To investigate how targeted systems affect the contact time, this paper records the variation of *t*_c_ in a wide range of *We* and *θ*_Y_, and the relevant results are shown in **Fig 6**. Simulation results show that the contact time is strongly dependent on *We* at lower impact velocities (*We* < 50, see **Fig 6**). However, the variation of *t*_c_ has a weak correla*t*ion with the intrinsic wettability. Therefore, the increase in *We* must result in a rapid decrease in the contact time (*We* < 50, see **Fig 6**) because more kinetic energy can be stored in spreading droplets to promote bouncing behavior. Except for high *We* range, the impingement dominated by inertia force determines that the spreading behavior as well as the varying *t*_c_ are out of con*t*rol by the intrinsic wettability. However, it is regrettable to demonstrate that there is a theoretical limit of *t*_c_ for impacting binary drople*t*s so that further increasing *We* cannot reduce the *t*_c_, as shown in **Fig 6**. The mechanisms underlying this are *t*he same as the impingement of the single droplet. The maximum spreading diameter of droplet after collision with a solid surface is determined by the balance of inertial and capillary forces, and the deformation over spreading increases with the increasing *We*. The spreading morphology of the droplet gradually transforms from a Hertzian sphere to a thin-film state. And the spreading water film is more likely to be an analogous non-Newtonian fluid with a certain degree of rigidity under this case. Furthermore, the constant contact time appears to be similar to the macroscopic phenomenon, but the intrinsic mechanism is very opposite [46]. The viscous force is non-negligible at the nanoscale. The viscous dissipation of the spreadingt process increases continuously along with increasing *We*, which inhibits the retraction of the merged droplets, and ultimately leads to the contact time remaining stable in a certain *We* range.

**Fig 7:**
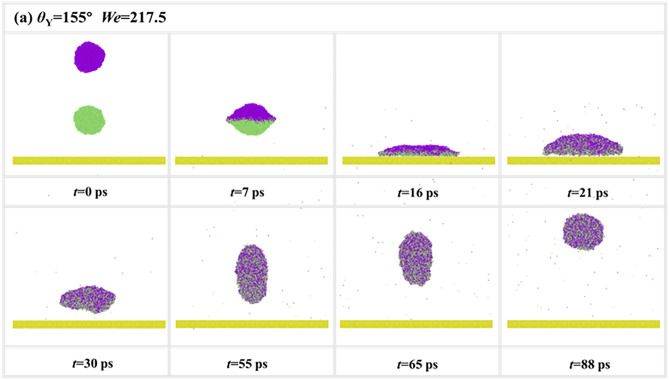
Especial dynamics of impacting binary nanodroplets for breaking theory limitation of the contact time on superhydrophobic surface at *We* = 217.5.

Compare to single droplets, the contact time reveals the unique differentiation, as shown in **Fig 6b**. Ref. [52] explains the inverse increase in contact time for single droplets at high *We*. Due to the creation of holes from the vibration of the central film, more kinetic energy is converted to surface energy during the retraction stage, increasing the contact time. Remarkably, the impingement of binary nanodroplets enables further reduction of *t*_c_ to reach another limitation value at high *We* range. In addition, the increasing Young contact angles can significantly reduce the critical value for inducing the special theory limitation (i.e., the second limitation). To understand the mechanism enabling impacting binary droplets to surpass the conventional contact time limit, the section examines the instantaneous evolution depicted in **Fig 7**. The intrinsic wettability of the solid surface is selected as *θ*_Y_ = 155°, and the impact *We* is 217.5. The snapshots reveal a highly energetic and rapid coalescence process, leading to the formation of an extremely thin spreading film. Next, the merged droplet forms the bulging liquid interface at *t* = 16 ps. Up to this point, the initial evolution is conventional and similar to that observed on moderately hydrophobic surfaces (**Fig 4b**). For the superhydrophobic surface, it is observed that the merged droplet can quickly bounce off from the solid surface. This rebound mode is very similar to the pancake rebound that Ma et al. have found to occur for nanodroplets under ultrahigh hydrophobicity in Ref. [53], where the bounce occurs at the early retraction stage so that the droplet can not complete the full retraction (see *t* = 21 ps), thus further reducing the contact time. The convex liquid deformation retracts in vacuum and gradually becomes a strange shape with elongated deformation along the x-direction. The elongated bouncing droplet experiences ongoing retraction; ultimately, the bouncing droplet can reduce the deformation spontaneously to recover the spherical shape, see *t* = 88 ps.

**Fig 8:**
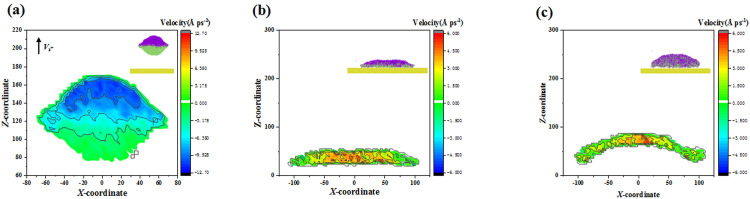
The velocity distribution within merged droplet up on surface with *θ*_Y_ = 155° at *We* = 217.5 at (a) *t* = 7 ps, (b) *t* = 16 ps, and (c) *t* = 21 ps.

**Fig 9:**
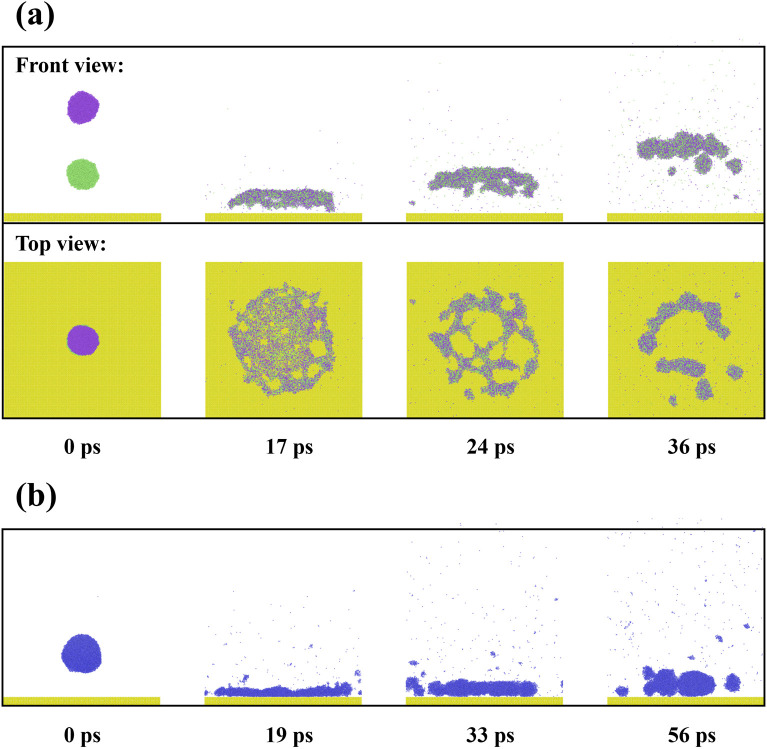
The breakup dynamics of (a) impacting binary droplets and (b) single droplet with controlling volume condition on superhydrophobic surfaces at *We* = 300. 24, and *We* = 170. 56.

To reveal the mechanisms underlying this, this paper extracts the velocity distribution within the merged droplet at *t* = 7 ps, 16 ps, and 21 ps, as shown in **Fig 8**. After the merged droplet forms, there shows an obvious velocity gradient is shown between the upper and lower parts, see **Fig 8a**. Subsequently, the merged droplet starts to spread upon the surface and attains its maximum deformation. For the conventional recognition, the droplet at the maximum spreading state can consume all the kinetic energy, except for some of which are dissipated by viscous dissipation. However, for impacting binary droplets, not all the kinetic energy is used to droplet spreading, so that the uneven velocity gradient exists throughout the whole coalescence-spreading process, see **Fig 8b**. Additionally, the intimate contact between the droplet and the solid surface quickly changes the direction of velocity from downward to upward. Therefore, the merged droplet with higher velocity concentrated in the middle part rises itself up (see **Fig 8c**) to form the special bouncing dynamics on the superhydrophobic surface to break down the first theory limitation of *t*_c_. This fascinating phenomenon has never been reported before and only occurs for impingement of the suspending droplet using a coming one, driven by the uneven velocity gradient. As the progressive increase in *We*, a different view starts to emerge, and its dynamic process has been illustrated in **Fig 9a**. The intrinsic contact angle is also selected as *θ*_Y_ = 155°, and the impact *We* here is in an extremely high value of *We* = 300.24. The merged droplet rapidly forms a thin film resembling its maximum spreading state even before contacting the solid surface (second snapshot in **Fig 9a**). The merged droplet escapes from the surface in a plump shape with the generation of some holes, which makes the bouncing droplet collapse into many fragments, i.e., breakup of impacting binary nanodroplets. Finally, these tiny fragments can coalesce and join to several daughter droplets (**Fig 9a**). Generally speaking, the breakup is one of the representative dynamics for impacting a single droplet. For the breakup dynamics of a single impacting droplet, the paper find that the critical value for inducing rebound is much lower compared with the present style, as shown in **Fig 9b**. This is because the collision of two droplets may generate the capillary-wave effect, which indeed consumes an amount of initial energy [54]. Hence, the critical *We* for breakup behavior of impacting binary droplets may be postponed to a large value.

## 4. Conclusions

In this work, MD simulations have been used to investigate the impact behavior of suspending droplets upon solid surfaces at the nanoscale by coming one. Simulation of intrinsic wettability is selected over a wide range from hydrophilic to superhydrophobic, and the scale of impacting *We* is from about 0.3 to 300.

The dynamic evolution of impacting binary nanodroplets has been concluded for different given conditions. Two separated droplets are initiated with the primary spreading, i.e., forming a completely merged droplet due to the pressure difference. The merged droplet then undergoes the usual spreading behavior (secondary spreading), driven by the rest of kinetic energy, as that for an impacting single droplet. Finally, the droplet deposits upon solid surfaces to form a wettable state, reaching an equilibrium between capillary and cohesive forces. As *We* increase, the obvious evolution for both primary spreading and secondary spreading can be observed. A very thin spreading film can be observed over the primary spreading and even formation of hole spreading for the secondary spreading at an extremely high *We* range. The scalar law of the maximum spreading factor is found to differ from the macrosystems through the data extracted by MD simulations. Since the velocity exists among the whole impacting droplet, so the maximum spreading factor over the primary spreading follows *β*_max, pre_ ~ *We*^1/2^. Whereas, for the secondary spreading factor, the substrate-induced velocity gradient triggers an additional viscous dissipation leading to the obeying of *β*_max, sec_ ~ *We*^1/2^*Re*^1/5^. The impingement is located in the capillary regime at the low *We* range, which could convert to the inertial regime as a progressive increase in *We*, and hence, the wettability of solid surfaces only affects the spreading factor at the low *We* range.

Significantly, for impacting binary nanodroplets, the contact time *t*_c_ exhibits dual theoretical minima, representing a key finding. The first limitation is very common in the impacting single droplet, where the droplet spreads from a Hertzian sphere to a thin-film state as *We* increases, gradually reaching a plateau period. Strikingly, the second limitation occurs only in special systems, where the asymmetric velocity gradient within the merging droplet concentrates the kinetic energy in the central region, enabling ultrafast bounce without the need for full retraction. This mechanism can reduce *t*_c_ below the classical threshold, offering a novel approach for applications requiring rapid droplet detachment such as anti-icing. In addition, the non-uniformly distributed energy within the droplet leads to a much larger breakup critical *We* for binary droplet collisions than for single system, providing ideas for some specific production.

## Supporting information

S1 File Complete data file. (ZIP)
